# Between-subject similarity of functional connectivity-based organization of the human periaqueductal gray related to autonomic processing

**DOI:** 10.3389/fnins.2022.1028925

**Published:** 2022-10-19

**Authors:** Mathijs M. de Rijk, Janine M. W. Janssen, Susana Fernández Chadily, Lori A. Birder, Mohammad S. Rahnama’i, Gommert A. van Koeveringe, Job van den Hurk

**Affiliations:** ^1^Department of Urology, School for Mental Health and Neuroscience, Faculty of Health, Medicine and Life Sciences, Maastricht University, Maastricht, Netherlands; ^2^Department of Urology, Maastricht University Medical Center+ (MUMC+), Maastricht, Netherlands; ^3^Department of Medicine, University of Pittsburgh School of Medicine, Pittsburgh, PA, United States; ^4^Scannexus Ultra-High Field MRI Center, Maastricht, Netherlands

**Keywords:** interoception, autonomic regulation, parcellation, brain mapping, brain stem

## Abstract

The periaqueductal gray (PAG) is a brain stem area designated to play an essential role in lower urinary tract (LUT) control. Post-mortem human and animal studies have indicated that the PAG is symmetrically organized in functionally and anatomically distinct columns which are involved in sympathetic or parasympathetic autonomic control of the LUT. The current study aims to find consistency across subjects and identify homologous clusters between subjects. Here, we evaluated data from 10 female participants. During a bladder filling protocol, we ran a resting-state functional magnetic resonance imaging (fMRI) scan while participants experienced a strong desire to void. A voxel-by-voxel correlation matrix of the PAG was created and parcellated using the Louvain module detection algorithm. Resulting in a map of the PAG in which each voxel is assigned to a cluster as determined by the Louvain module detection algorithm. The spatial similarity of resulting clusters between participants was assessed by computing the Dice similarity coefficient for all cluster comparisons. Next, we designed a permutation test to create randomized parcellation maps which enabled us to statistically test the similarity values observed across participants. We observed several significantly similar clusters between subjects compared to permutations (*p* ≤ 0.05). These results show that the PAG can be parcellated into distinct clusters which show a similar spatial distribution at the group level. This analysis is a crucial step to determine the agreement between *in vivo* PAG parcellations and the functional and anatomical columnar organization of the PAG which is known from previous research. These advancements may enable us to identify the relationship between LUT symptoms, such as urgency, and activity patterns in the PAG in normal and pathological states.

## Introduction

There is a continuous exchange of information between the peripheral nervous system (PNS) and central nervous system (CNS) regarding the internal state of the body. Most of the time these processes do not reach our conscious awareness, but our attention will be shifted to visceral functioning when needed in order to maintain a proper homeostatic balance (Craig, 2002). The spinal cord and brain stem have key functions in relaying interoceptive information from peripheral organs to higher brain areas. That way, these structures serve a crucial role in the bidirectional transfer of visceral information to our awareness and to the autonomic nervous system for subconscious regulatory purposes (Critchley and Harrison, 2013). Proper exchange of information between the CNS and PNS is essential for the regulation of autonomic processes and dysfunction of this system can present in disturbances in nociceptive, cardiovascular, respiratory, urinary, gastrointestinal, and thermoregulatory functions.

Storing and voiding of urine is regulated by a delicate interplay between visceral sensations regarding bladder fullness and socially learned behavior concerning appropriateness of emptying one’s bladder at a certain place and time. Therefore, the control of the bladder and urinary sphincter is arguably one of the most evident visceral functions that is highly dependent on multifaceted processes that require complex and coordinated activity, and integration of peripheral afferent and central efferent signals at different levels (Birder et al., 2010). The control of the lower urinary tract (LUT) is hierarchically organized within the CNS and the PNS. In healthy adults, bladder sensory processing and the initiation of micturition is controlled by dedicated areas at the level of the spinal cord [mainly Onuf’s nucleus (ON)], the brain stem [e.g., pons and periaqueductal gray (PAG)], and higher cortical and subcortical areas (e.g., prefrontal cortex, anterior cingulate cortex, and insula) (Fowler et al., 2008).

The PAG is a brain stem structure surrounding the cerebral aqueduct and is approximately 14 mm in length. The PAG is an integral part of the emotional motor system and as such is highly involved in a wide array of processes related to nociceptive control, cardiovascular control, respiration, micturition, defecation, parturition, and many more (Holstege, 2016). The PAG is centrally located in the hierarchical system of micturition control, and is assumed to serve as a relay station projecting afferent information from the bladder to cortical and subcortical brain areas and as a gatekeeper projecting efferent information from these higher areas to the pontine micturition center (PMC) and ON (Blok et al., 1995, 1998; de Groat et al., 2015; Zare et al., 2019). Animal research has indicated that the PAG is organized in a symmetrical columnar fashion, and the ventrolateral and dorsolateral areas of the PAG are indicated to be involved in the control of voiding and storage of urine, respectively (Noto et al., 1989; Liu et al., 2004; Numata et al., 2008; Meriaux et al., 2018; Zare et al., 2018).

Previous functional neuroimaging research has identified some of the pathways and has indicated that the insula and anterior cingulate cortex, known as the sensory and autonomic areas for the autonomic nervous system, are involved in processing bladder sensory information and LUT control (Griffiths and Tadic, 2008; Griffiths et al., 2009). The insula monitors bladder sensory information and, when necessary, shifts our attention to our bladder so that we can look for an appropriate time and place to initiate voiding. These higher level processes and the ultimate decision to void require frontal cortex activity (Gao et al., 2015; Coolen et al., 2020; Locke et al., 2022). These cortical and subcortical structures are likely to exercise their control over the LUT *via* the PAG.

Optimal functioning of these central systems enables healthy adults to accurately assess their experienced levels of bladder fullness at any given time and to reliably predict for how long they will be able to postpone micturition. In conditions of LUT dysfunction, such as overactive bladder (OAB), participants might struggle with these tasks. The International Continence Society defines OAB as urgency, with or without urge incontinence, and often with frequency and nocturia. Over the past years, it has become more apparent that OAB is associated with altered bladder sensations. Mechanisms of central sensitization might underlie bladder hypersensitivity and sensory disturbances leading to alarm falsification (Reynolds et al., 2016). A disturbance in the neural processing of visceral sensory information might be related to this type of LUT dysfunction. Recent neuroimaging work has reported the first indications that PAG activity reflects subjective reported bladder sensations (de Rijk et al., 2021). PAG activity might, therefore, offer insights into processing of visceral sensory information and could help identify alterations in interoceptive processes which might cause “false alarms” in patients with OAB.

Previous research has indicated that the PAG can reliably be subdivided into distinct clusters of functional regions that can be differentiated based on resting-state functional magnetic resonance imaging (fMRI) data at 7 Tesla (T). At the within-subject level, these clusters show a symmetrical organization and high level of similarity between empty and full bladder states (de Rijk et al., 2021), which is in line with what would be expected based on animal work.

In the current study we aimed to assess the similarity between PAG organization at the group level using resting-state fMRI at 7T. We expected to observe a significantly higher spatial overlap between clusters from different participants than would be expected based on chance.

## Materials and methods

### Participants

This study was designed and conducted in line with the Declaration of Helsinki and was approved by our local ethics committee. Written informed consent was obtained from each of our participants. Participants provided consent for untraceable use of their functional and structural MRI data. We enrolled 13 female participants in our study (mean age: 46.31, range: 21–73) without any clinically significant history of LUT dysfunction, or neurological disease or dysfunction, as judged by the medical investigator. We chose to include only female participants in this project to control for gender as a confounding factor. We had to exclude data obtained from 3 participants due to technical difficulties. All participants were able to complete all steps of the bladder filling and scanning protocol.

### Study design

After obtaining informed consent, participants were asked to fill out a 3-day micturition diary in order to gain baseline values of their voiding- and drinking patterns and to familiarize the participants with scoring their perception of urgency on the 4-point Indevus Urgency Severity Scale (IUSS) (Nixon et al., 2005).

Participants were then invited to visit our clinic for the fMRI study. Upon arrival at our institute participants were instructed to void until empty in private after which a nurse inserted a filling catheter (FR: 8) transurethrally. Any residual urine was emptied from the bladder *via* this catheter. Participants were then instructed to lie down in a supine position on the MRI bed while an MRI-compatible syringe pump was connected to the filling catheter. Head motion was restricted using foam cushioning. Scanning was conducted on a 7T MRI scanner (Siemens, MAGNETOM) using a 32-channel head coil (NOVA Medical, Wilmington, MA, USA).

First, we ran a T1-weighted whole brain anatomical scan using an MP2RAGE sequence. We then manually prefilled the bladder with a syringe at a rate of 30 ml/min to 50% of the volume of the micturition episode with the smallest volume reported on the micturition diary with a score of 1 on the 4-point IUSS (range 0–3) in order to decrease individual differences in total filling time. The bladder was then further filled using an automatic syringe pump at a speed of 30 ml/min. Participants were instructed to report their experienced levels of urgency, using a joystick they could control from the scanner, on the IUSS for their perceived levels of urgency. Reported bladder sensations were visually presented to the researcher in the operating room in real-time and were logged every second using a custom written MATLAB script. Once participants indicated urgency levels of 2 on the 4-point IUSS, defined by Nixon et al. (2005) as “enough urgency discomfort that it interferes with or shortens your usual activity or tasks” (p. 607), the bladder filling protocol was stopped and a full bladder resting-state scan was started.

During this resting-state scan we collected 420 T2*-weighted multiband echo planar imaging volumes (mb-EPI sequence, acceleration factor = 2, MB-factor = 2, TR = 1,400 ms, TE = 22 ms, resolution = 1.1 × 1.1 × 1.1 mm). Participants were instructed to keep their eyes open during this scan, and try not to think of anything in particular. We scanned 40 slices covering the supramedullary portion of the brain stem. After the full bladder scan was finished, the participants were assisted out of the scanner and instructed to void until empty in private while recording their voided volume.

### Data processing

Functional data were preprocessed using BrainVoyager 22.2 (Brain Innovation, Maastricht, The Netherlands). Functional volumes were first corrected for slice scan-time differences and 3D head motion using 3 translation and 3 rotation parameters. Subsequently, linear trends and low frequency temporal drifts were removed from the data using a high-pass filter, removing temporal frequencies below 0.1 Hz, which is suggested to be an appropriate threshold for preservation of functional connectivity information in the data (Margulies et al., 2010; Yeo et al., 2011). Functional data were then co-registered to the anatomical images. The whole brain anatomical images were transformed to MNI space, after which the functional data was transformed to MNI space using the same parameters as the structural data.

### Periaqueductal gray parcellation

We identified and segmented out the PAG in the MNI template using BrainVoyager 22.2. The mask file resulting from this approach contained 1,169 voxels within the region of interest. For the full bladder resting-state fMRI scans of each participant, the time course of each voxel within the PAG mask in MNI space was selected and correlated in a pair-wise fashion, to obtain a voxel-by-voxel symmetric square connectivity matrix. We removed the lowest 5% correlations that are assumed to represent spurious connections from our matrix (Rubinov and Sporns, 2010).

To generate parcellation maps of the PAG, we partitioned the connectivity matrices of the full bladder resting-state data into clusters using a MATLAB implementation of the Louvain module detection algorithm (Blondel et al., 2008). This algorithm outputs modules with stronger within-module connectivity than between-cluster connectivity (Fortunato, 2010) without the need for a predetermined number of clusters. The assignment of voxels to modules by the Louvain module detection algorithm has a stochastic nature, and in order to compensate for this we ran the algorithm for 500 iterations and selected the parcellation with the largest modularity value (*Q*-value) for further analysis. The *Q*-value quantifies the extent to which a network has a modular underlying data structure (Girvan and Newman, 2002). Our parcellation algorithm then assigned a label (a number between 1 and the total number of clusters) to each voxel in the PAG allocating it to a module. These labels were then used to create a module map for each participant for full bladder datasets.

### Cluster matching across participants

We expect that if our parcellations reflect an underlying physiological construct present in the general population we should be able to observe spatially highly similar clusters across participants. We assessed the similarity between PAG parcellations across participants and statistically tested our observations by designing a permutation test.

The similarity of clusters resulting from the parcellation procedure between participants was assessed by computing the Sørensen–Dice similarity coefficient for all cluster comparisons across participants. The Sørensen–Dice coefficient is a metric that assesses spatial overlap by multiplying the area shared by both datasets by 2 and subsequently dividing this number by the total area of both datasets combined. In this way the Sørensen–Dice coefficient is a useful measure to assess the similarity between two images.

Next, we designed a custom MATLAB script in which, for each iteration, we randomly picked a subject’s correlation matrix and shuffled the observed correlation values across the matrix. Subsequently, this code created a randomized symmetric correlation matrix reflecting the same range of correlation values as the original data. We iterated this script in order to generate 1,000 randomized correlation matrices which were subsequently parcellated using the Louvain module detection algorithm in the same approach as the original data. From these 1,000 randomized PAG parcellation maps we computed the Sørensen–Dice coefficient for 100,000 randomly selected cluster combinations between maps in order to obtain a distribution of Sørensen–Dice coefficients under the null hypothesis.

We then statistically assessed the significance of the original Sørensen–Dice coefficients between cluster pairs from different participants by ranking them to the distribution of Sørensen–Dice values observed in the permutations after correcting the false discovery rate for multiple comparisons.

## Results

**FIGURE 1:**
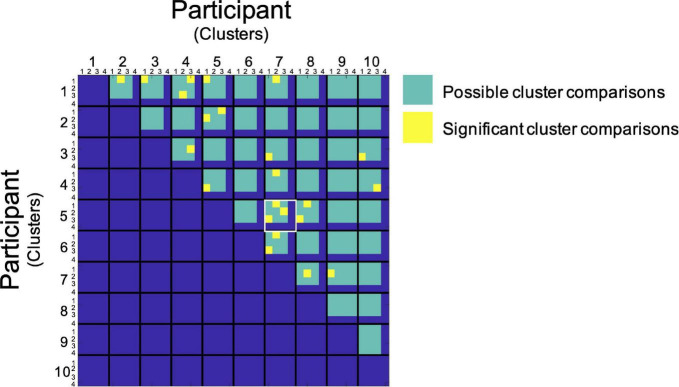
Group level assessment of spatial similarity of PAG clusters across participants. Data from each participant are represented in a single cell. Along the x and y direction comparisons between subjects and clusters are plotted. Subjects are not compared with themselves and only in one direction. Each yellow block marks a statistically significant similarity between clusters [*p* ≤ 0.05, after FDR corrections (*q* = 0.05)]. The green area indicates the comparisons made (the Louvain detection algorithm parcellated some PAGs into 4 clusters and others into 3 clusters). The comparison between participants 5 and 7 has been highlighted in white since all clusters from these two participants showed significantly high similarity.

**FIGURE 2:**
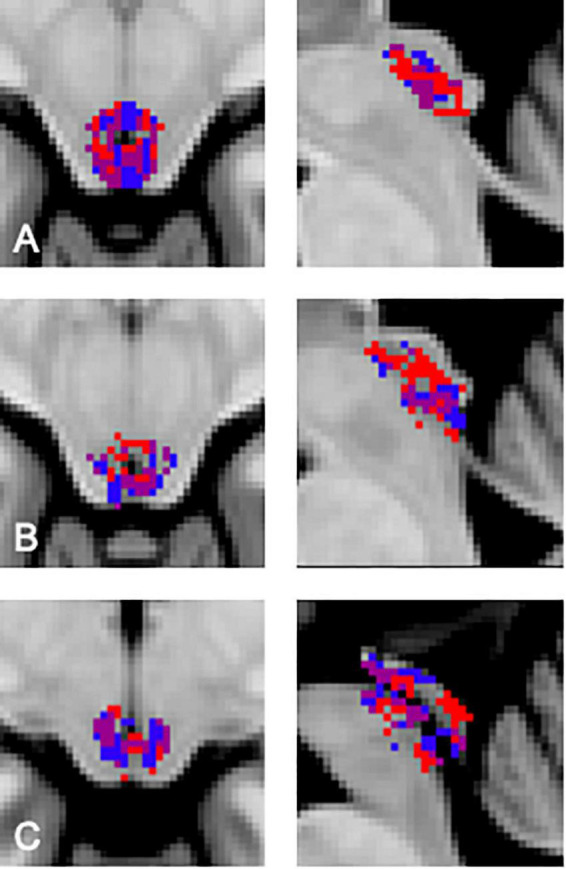
Similarity between the different clusters from participants 5 and 7. The purple area indicates the region of overlap between participants. **(A)** Transversal and sagittal view of the overlap between participant 5, cluster 1 (red) and participant 7, cluster 2 (blue). **(B)** Transversal and sagittal view of the overlap between participant 5, cluster 2 (red) and participant 7, cluster 3 (blue). **(C)** Transversal and sagittal view of the overlap between participant 5, cluster 3 (red) and participant 7, cluster 1 (blue).

In order to visualize the functional organization of the human PAG we determined the consistency of resting state functional connectivity based parcellations across participants. Data from each participant was transformed to the MNI template in order to be able to include the same voxels for each participant in our analyses. After successful parcellation of PAG connectivity matrices for all participants, we assessed the similarity between PAG parcellation maps by comparing Sørensen–Dice coefficients observed between cluster combinations across participants to values expected under the null hypothesis as determined by our permutation test. In this permutation test, the similarity of blood-oxygen-level-dependent (BOLD) signal fluctuations of voxels based on spatial proximity and functional connectivity was randomized across the matrix in order to generate parcellation maps based on data without topographical data regarding PAG organization, but with maintenance of correlation values corresponding to the original data. We found that for each of our participants the agreement between at least one of the clusters, resulting from the resting-state fMRI parcellation procedure, with a cluster from another participant was significantly higher than could be expected based on the empirical distribution under the null hypothesis resulting from our permutation test [*p* ≤ 0.05, corrected for multiple comparisons using a false discovery rate (*q* = 0.05)]. We observed a significantly higher similarity between cluster pairs across subjects compared to permutations. For 23 cluster combinations across participants we observed a significantly higher similarity than could be expected based on the distribution under the null hypothesis (Figure 1). Between two of our participants (participant 5 and 7, Figure 1) we found a significant match for all clusters (Figure 2). Additionally, there seems to be a higher order consistency between clusters across participants. For example: There is a significant similarity between participant 1—cluster 1 and participant 2—cluster 2 (Figure 1), as well as between participant 1—cluster 1 and participant 5—cluster 1. Moreover, the similarity between participant 2—cluster 2 and participant 5—cluster 1 is significantly higher than would be expected based on the distribution under the null hypothesis as well (Figure 1). This suggests that the functional organization of the PAG, as determined by parcellating this structure using the Louvain module detection algorithm in MNI space, shows measurable consistency at the group level.

## Discussion

Here, we aimed to use 7T fMRI data acquired during a full bladder state to functionally subdivide the human PAG into clusters which show consistency at the group level. Our findings show that parcellating the PAG using the Louvain module detection algorithm resulted in clusters that show significant agreement across subjects. The reported results improve our understanding of the functional organization of this small brain stem structure and enable assessment of the role specific topographical PAG regions have in the control of the LUT.

We have previously assessed some of the known anatomical characteristics regarding anatomical organization of the PAG and have shown that resting-state fMRI based parcellations result in clustering which show symmetrical organization as well as consistency between empty and full bladder states in healthy subjects (de Rijk et al., 2021). The results we report here, additionally, show that the PAG can be subdivided into distinct functional clusters with a similar spatial distribution across participants in full bladder states. These findings provide additional support that full-bladder resting-state fMRI based PAG parcellations show characteristics which are in line with organizational characteristics known from earlier neuroanatomical studies.

Since the 1990’s there is a general consensus that functionally distinct areas within the PAG are arranged in symmetrical longitudinal columns extending along the rostrocaudal axis of the PAG (Bandler and Shipley, 1994; Depaulis and Bandler, 2012). The PAG is assumed to be organized in three functionally and histologically distinct pairs of columns: the ventrolateral, lateral, and dorsolateral columns, and a single dorsomedial column. Using diffusion MRI, researchers have previously segmented the PAG into columnar modules using tractography-based segmentation methods (Ezra et al., 2015). This method uses the diffusion direction of water molecules to group together voxels in a region with similar diffusion properties. Along the cerebral aqueduct this resulted into clusters showing a columnar organization along the rostrocaudal axis of the PAG, with spatial similarity to animal models. Investigation of connectivity patterns for the different columns with predefined cortical and subcortical target regions, based on previous animal and human studies, showed unique connectivity patterns for the different columns (Ezra et al., 2015).

On visual inspection, the functional clusters observed in our parcellation maps do not evidently show the columnar organization that one might expect to see based on animal research and post-mortem human studies. We argue that the nature of the resting-state fMRI signal during an awake full bladder state in humans reflects a fundamentally different signal from animal and post-mortem work. It is known that, to some extent, there are regional differences in somatotopic and viscerotopic organization along the rostrocaudal axis within individual PAG columns. Additionally, neuroanatomically distinct columns appear to have partially overlapping functional areas (Depaulis and Bandler, 2012). The awake and alert full bladder state in which our participants were scanned, with continuously ongoing autonomic processes, might result in PAG parcellation maps consisting of functional clusters that are in an actively recruited state to execute these processes and show high correlations in their activity which might reflect communication across columns or within restricted areas along the rostrocaudal axis. The exact nature of the relationship between clusters resulting from resting-state fMRI based parcellations and the columnar neuroanatomical organization of the PAG requires further research to fully be elucidated. Analysis of brain stem functional connectivity patterns investigating different characteristics of resting-state fMRI, such as regional homogeneity and amplitudes of low-frequency fluctuations, may be useful future approaches.

By the assessment of functional connectivity patterns between voxels within the PAG we have been able to group together voxels that show a high similarity in their blood-oxygen-level-dependent signal during a full bladder state. This provides a new way to functionally map this small brain stem structure without the need for *a priori* assumptions regarding functional anatomy of this small structure. The advantage of the method presented in the current paper is the largely data driven approach of parcellating the PAG, without the need for a predetermined number of clusters or hand drawn constraints for the shape and orientation of clusters. Once PAG parcellation maps based on resting-state functional connectivity patterns are established, future research should investigate dynamic changes in functional connectivity patterns within this region, as well as with cortical and subcortical areas involved in LUT control, during manipulation of bladder sensory processing.

Neuroimaging data inherently contains some level of signal noise. We argue that the permutation test we designed enables us to assess whether the observed similarity between cluster combinations can be explained solely by signal noise. The cluster combinations that were significantly more similar than would be expected under the null hypothesis, resulting from the permutation test, give an indication of the reliability and reproducibility of the reported results and suggest that the significantly high dice similarity coefficients between clusters across participants cannot be explained by signal noise alone. The small sample size, focus on female participants, and relatively large age distribution of participants puts limits to the results of the current study However, the analysis shown here is a crucial step to determine the agreement between *in vivo* fMRI based PAG organizational maps and the functional and neuroanatomical organization of the PAG which is known from previous research. These advancements are essential in order to enable us to identify the relationship between LUT symptoms, like urgency, and activity patterns in the PAG in normal and pathological states. Establishing this relationship will allow determination of interindividual conformity or diversity.

Further investigation of how CNS activity patterns relate to subjective bladder fullness and urgency sensations can lead to identification of fMRI imaging biomarkers regarding OAB. A further unraveling of mechanisms such as alarm falsification in OAB could potentially lead to new, non-invasive therapies like interoceptive bladder awareness training *via* bio-feedback. Moreover, this research may help us understand the underlying mechanisms of current therapies, including sacral neuromodulation, and improve patient selection strategies.

To conclude, the PAG is an important brain stem nucleus involved in sensation and control of the LUT and various other visceroceptive processes. At the within-subject level, resting-state fMRI data of the PAG can be used to subdivide this nucleus into clusters that show some of the anatomical characteristics known from animal and post-mortem studies. Here, we show that PAG clusters additionally show high spatial organizational similarity at the group level. This paves the way for analysis of PAG activity related to bladder sensation and control at the group level, as well as studies aiming to unravel the interaction between the PAG and the rest of the brain. Utilizing these approaches to study CNS changes in response to successful therapeutic interventions will not only help to improve current therapies and patient selection strategies, but also may lead to the development of new therapies.

## Data availability statement

The raw data supporting the conclusions of this article will be made available, without undue reservation, upon request to the corresponding author.

## Ethics statement

The studies involving human participants were reviewed and approved by METC AzM/UM. The patients/participants provided their written informed consent to participate in this study.

## Author contributions

MR, JJ, SF, LB, MR, GK, and JH: conception and study design. MR, JJ, and SF: data collection or acquisition. MR and JH: statistical analysis. MR, JH, and GK: interpretation of results. MR: drafting the manuscript. MR, JJ, SF, LB, MR, GK, and JH: revising it critically for important intellectual content. All authors approved the final version to be published and agreed to be accountable for the integrity and accuracy of all aspects of the work.
